# Draft Genome Sequences of Four Lactococcus lactis Strains Isolated from Diverse Niches, Including Dairy Products, Grass, and Green Peas

**DOI:** 10.1128/MRA.00834-19

**Published:** 2019-08-29

**Authors:** Desirée Roman Naranjo, Michael Callanan, Olivia McAuliffe

**Affiliations:** a Teagasc Food Research Centre, Fermoy, Cork, Ireland; b Cork Institute of Technology, Cork, Ireland; University of Maryland School of Medicine

## Abstract

Lactococcus lactis has been used for millennia as a starter organism in the production of many fermented dairy products. This announcement includes the draft genome sequences of four strains of Lactococcus lactis, two of dairy origin and two from nondairy sources.

## ANNOUNCEMENT

Lactic acid bacteria (LAB) are widely used as commercial dairy starter cultures. Within this group, Lactococcus lactis is the main species used as an industrial and artisanal starter culture due to its ability to produce acid from lactose in milk and to generate a diverse range of flavors (1). Even though this species is traditionally associated with the milk environment, the ancestor of common L. lactis strains occupied a plant niche (2), and indeed, L. lactis possesses a wide ecological distribution in diverse niches. In this context, L. lactis strains can be classified as “domesticated” (related to dairy and fermented products) or “environmental” (isolated from plants, animals, and raw milk) (3). Recently, nondairy L. lactis strains have gained some attention because of their more diverse metabolic capabilities and flavor-forming capacities not usually found in dairy strains (4, 5).

Whole-genome sequencing was performed for two domesticated strains, Lactococcus lactis subsp. *cremoris* DPC 140 and Lactococcus lactis subsp. *cremoris* DPC 169, both isolated from mixed-strain starter cultures, and for two wild, or nondairy, strains, Lactococcus lactis subsp. *lactis* DPC 6756 (strain P-8 described by Alemayehu et al. [5]), isolated from green peas, and Lactococcus lactis subsp. *cremoris* DPC 6855 (6), isolated from grass. Dairy strains were grown overnight in M-17 broth (Sigma-Aldrich) at 30°C. Nondairy strains were supplemented with 0.5% (wt/vol) d-glucose (Sigma-Aldrich). DNA was isolated using the UltraClean microbial DNA isolation kit (Mo Bio Laboratories, Cambridge, UK) following the manufacturer’s instructions. Default parameters were used for all software unless otherwise specified. Genomic DNA libraries were prepared using a Nextera XT library prep kit (Illumina, San Diego, USA) following the manufacturer’s protocol with the following modifications: 2 ng of DNA instead of 1 ng was used as input, and the PCR elongation time was increased from 30 s to 1 min. Libraries were sequenced on the Illumina HiSeq platform using a 250-bp paired-end protocol (MicrobesNG, University of Birmingham, UK). Reads were adapter trimmed using Trimmomatic 0.30 with a sliding window quality cutoff value of Q15 (7). SPAdes 3.7 was used to perform *de novo* assembly on the samples (8). The genome sequences were annotated using the NCBI Prokaryotic Genome Annotation Pipeline (PGAP) (9). The final draft genomes were estimated using CheckM 1.0.12 (10) to be ≥96% complete with <2.5% contamination.

The sequencing data and statistics of the above-mentioned strains are shown in Table 1. These sequencing data contribute to the pool of available Lactococcus lactis genomes from diverse sources for further analysis of potential phenotypes and genotype-phenotype associations. Metabolic pathways can be explored and compared across niches in order to discover new capabilities of interest in the food industry, such as enzyme production and flavor formation.

**TABLE 1:** Genome characteristics of the Lactococcus lactis strains used in this study

Organism	Draft genome size (bp)	No. of contigs	*N*_50_ (bp)	G+C content (%)	Mean coverage (×)	Total no. of reads	SRA accession no.	GenBank accession no.
*L. lactis* subsp. *cremoris* DPC 140	2,393,364	229	17,974	35.60	109	627,757	SRR9283149	VERZ00000000
*L. lactis* subsp. *cremoris* DPC 169	2,480,405	130	36,700	35.64	117	723,132	SRR9283148	VERY00000000
*L. lactis* subsp. *lactis* DPC 6756	2,482,547	13	637,420	34.96	83.3	497,681	SRR9283151	VERX00000000
*L. lactis* subsp. *cremoris* DPC 6855	2,694,688	101	151,720	35.37	37.9	253,524	SRR9283150	VERW00000000

### Data availability.

The draft whole-genome sequencing data were deposited in NCBI GenBank and the Sequence Read Archive (SRA) under BioProject no. PRJNA546489. The accession numbers are listed in Table 1.
